# How to Obtain a Degree or Diploma in Public Health

**Published:** 1911-09-02

**Authors:** 


### HOW TO OBTAIN A DEGREE OR DIPLOMA IN PUBLIC HEALTH.

By a D.P.H.

Without going into the merits of the public health service as a career, we may safely say that the possession of a degree or diploma in State medicine, sanitary science, or public health is an advantage which at the present time is widely appreciated. It is indispensable to candidates for any public health appointment, and has also become popular among many other classes of practitioners, such as those in the Poor-law and lunacy services, officers in the Army, Navy and Colonial services, and among general practitioners, especially those in districts where there is a chance of a small fever hospital appointment or a part-time school inspectorship. There is now no difficulty in obtaining the necessary courses of instruction, while there are some twenty-three diplomas and degrees conferred by the various universities and examining bodies.

A list of these may conveniently be given here:

| Examining Body. | Degree. |
|---|---|
| University of London | M.D. in State Medicine. |
| University of Oxford | D.P.H. |
| University of Cambridge | D.P.H. |
| University of Durham | B.Hy. (D.Hy.) and D.P.H. |
| University of Edinburgh | B.Sc. (D.Sc.) |
| University of Dublin | D.P.H. |
| Universty of Ireland ... | B.Sc., and D.P.H. |
| University of Aberdeen | D.P.H |
| University of Glasgow... | B.Sc. (D.Sc.) |
| University of St. Andrews | D.P.H. |
| University of Wales ... | D.P.H. |
| University of Belfast ... | D.P.H. |
| University of Birmingham | D.P.H. |
| University of Bristol ... | D.P.H. |
| University of Leeds ... | D.P.H |
| University of Liverpool | D.P.H. |
| University of Manchester | D.P.H. |
| University of Sheffield | D.P.H. |
| Conjoint Board of England ... | D.P.H. |
| Conjoint Board of Scotland ... | D.P.H. |
| Conjoint Board of Ireland ... | D.P.H. |

The regulations of these examining bodies are, in the main drawn up on similar lines, and their requirements and syllabuses do not differ very greatly. Restrictions are chiefly found in connection with the granting of degrees by some of the Universities to their own graduates, or require that certain portions of the courses of study shall be taken out in their own laboratories. Governing all the individual regulations are the resolutions adopted by the General Medical Council dealing with qualifications in this branch of medicine. These very important rules must be carefully studied, for they embody the essential requirements for the obtaining of such a degree or diploma, and we therefore make no excuse for setting them out here.

Rule 1.—A period of not less than twelve months shall have elapsed between the attainment of a registrable qualification in medicine, surgery, and midwifery and the admission of the candidate to any examination or any part thereof for a diploma in sanitary science, public health, or State Medicine.

Rule 2.—Every candidate shall have produced evidence that, after obtaining a registrable qualification, he has during six months received practical instruction in a laboratory or laboratories, British or foreign, approved by the licensing body granting the diploma, in which chemistry, bacteriology, and the pathology of the diseases of animals transmissible to man are taught.

Rule 3.—Every candidate shall have produced evidence that, after obtaining a registrable qualification, he has during six months (of which at least three months shall be distinct and separate from the period of laboratory instruction required under Rule 2), been diligently engaged in acquiring a practical knowledge of the duties, routine and special, of public health administration, under the supervision of—

(*a*) In England and Wales, the medical officer of health of a county or of a single sanitary district having a population of not less than 50,000, or a medical officer of health devoting his whole time to public health work; or

(*b*) In Scotland, a medical officer of health of a county, or counties, or of one or more sanitary districts having a population of not less than 30,000; or

(*c*) In Ireland, a medical superintendent officer of health of a district or districts having a population of not less than 30,000; or

(*d*) In the British Dominions outside the United Kingdom, a medical officer of health of a sanitary district having a population of not less than 30,000, who himself holds a registrable Diploma in Public Health; or

(*e*) A medical officer of health who is also a teacher in the department of public health of a recognised medical school; or

(*f*) A sanitary staff officer of the Royal Army Medical Corps having charge of an army corps, district command, or division, recognised for this purpose by the General Medical Council.

Note (1).—The certificate of an assistant medical officer of health of a county or of a single sanitary district having a population of not less than 50,000 may be accepted as evidence under Rule 3, provided the medical officer of health of the county or district in question permits the assistant officer to give the necessary instruction and to issue certificates.

Note (2).—Provided that the period of six months may be reduced to a period of three months (which shall be distinct and separate from the period of laboratory instruction required under Rule 2), in the case of any candidate who produces evidence that, after obtaining a registrable qualification, he has during three months attended a course or courses of instruction in sanitary law, sanitary engineering, vital statistics, and other subjects bearing on public health administration given by a teacher or teachers in the department of public health of a recognised medical school.

Note (3).—A candidate who shall have produced evidence that he has himself held for a period of not less than three years an appointment as medical officer of health of a sanitary district within the British Dominions, and having a population of not less than 15,000, may be exempted from the requirement of Rule 3.

Rule 4.—Every candidate shall have produced evidence that, after obtaining a registrable qualification, he has attended during three months the practice of a hospital for infectious diseases at which opportunities are afforded for the study of methods of administration.

Note (1).—Methods of administration shall include the methods of dealing with patients at their admission and discharge, as well as in the wards, and the medical superintendence of the hospital generally.

Note (2).—In the case of a medical officer of the Royal Army Medical Corps a certificate from a principal medical officer under whom he has served, stating that he has during a period of at least three months been diligently engaged in acquiring a practical knowledge of hospital administration in relation to infectious diseases, may be accepted as evidence under Rule 4.

Rule 5.—The examination shall have been conducted by examiners specially qualified; it shall have extended over not less than four days, one of which shall have been devoted to practical work in a laboratory, and one to practical examination in, and reporting on, subjects which fall within the special outdoor duties of a medical officer of health.

It will be gathered from the above that the course of study requires the expenditure of at least nine months. By attending lectures in sanitary law, etc., three months of the six to be spent in association with a Medical Officer of Health may be obviated, but these three months must be distinct from the six months which have to be spent in working in a laboratory. The three months' fever work may be taken at any time subsequent to becoming a registered medical practitioner, or may be done while taking the practical courses in the laboratories. The attendance at a fever hospital which is part of the medical student's curriculum will not count towards this for the D.P.H., as this instruction must be taken after qualification. Considerable latitude is allowed in the choice of a fever hospital, and local hospitals can often be conveniently made use of. It is well to write up to the examining board in question and ask for formal sanction for a certificate from any particular hospital. At two of the fever hospitals of the Metropolitan Asylums Board special courses of instruction are given at certain times of the year on purpose for D.P.H. candidates, and at which opportunities are afforded for the study of methods of administration. These hospitals are the North-Western and The Grove, and the fee for the course is three guineas. The lectures and demonstrations are given twice a week, but the student is only expected to attend once a week on the day most convenient to himself, for the lectures on the two days in any one week will be identical, one being given in the forenoon and one in the afternoon.

Of the rules given above, those numbered 2, 3, and 4 are not enforced in the case of a medical practitioner registered or entitled to be registered on or before January 1, 1890. Some modification of certain of these rules is contemplated by the General Medical Council, but this body has not yet finished its consideration of the points raised, so that no alterations are likely to come into force until after next year at the earliest.

In London a diploma is granted by the Conjoint Board of the Royal Colleges of Physicians and Surgeons after an examination conducted in two parts, the first of which must be passed before entering for the second part. The examinations are held twice a year—in January and July—and consist of written, oral and practical portions. The fee for each part of the examination is six guineas to holders of the Conjoint qualification and ten guineas to others. Candidates must give fourteen days' notice, and will have to be at least twenty-four years of age when sitting for the second portion of the examination.

Outside London, the Cambridge diploma is a very popular one, and is freely open to any qualified man or woman. The examination takes place in April and October, and the fee for each part is six guineas. Candidates are permitted to sit for the second part even if they have not passed or sat for the first; marks of distinction are now awarded to those who have done exceptionally well in any of the subjects of the examination. The syllabus and the general scope of work required are similar to those of the London examination, and the regulations are governed by the rules of the General Medical Council.

The State Medicine Syndicate approves of a large number of colleges and schools at which courses of laboratory instruction may be taken out, but instruction is given in the fine laboratories at Cambridge, and there is some advantage to be gained by doing the practical work at those laboratories.

The regulations at Oxford are similar to those in force at Cambridge, and a D.P.H. examination is held there twice a year. The fee is five guineas for each of the two parts, entry to which must be preceded by the sending in of certificates in accordance with the rules of the General Medical Council.

Generally speaking, it may be observed that candidates at most of the other centres have to comply with conditions so very similar that a detailed reference is unnecessary; but in some cases prescribed courses of lectures and laboratory work have to be taken out at the particular university in question, or the examination is confined to its own graduates. The University of Manchester not only grants a D.P.H. on these lines, but also gives certificates in school hygiene and factory hygiene to candidates who have attended the prescribed courses and have passed the special examinations. These certificates are only given to candidates who have obtained the diploma in public health.

The degree of B.Sc. in public health granted by several universities generally entails a wider course of study taken at the respective university, and is not often thrown open to others than their own graduates. The M.D. in State Medicine of the London University is, of course, only open to holders of the M.B., B.S. (Lond.).

Turning now to the subjects on which the candidate will be examined, it may be observed that they cover a very wide range, including a good deal of chemistry, physics and bacteriology, besides the more strictly sanitary and legal subjects, to say nothing of geology, meteorology and building construction. Studying the examination papers set in previous years is the best way of becoming acquainted with the scope of the examination. Water, sewage and foods are very important subjects, both in the papers and in the practical part. It would serve no useful purpose to enumerate here the details of the syllabus, for the prospective candidate will naturally apply for one to study for himself. Bacteriology is often considered a stumbling-block, but neither in the papers nor in the practical work is very advanced knowledge demanded. It is well not to forget to familiarise oneself with the appearances of the commoner parasites, not only of the human body but also those found in animals and food-stuffs; and here we may observe that every candidate should endeavour to obtain some practical experience in the inspection of meat at a large slaughter-house or at a public meat market. This may easily be arranged with one of the chief inspectors, who take pupils for the sanitary inspectors' examination.

In the second part of the examination for the D.P.H. the questions involve a practical knowledge of the work of a medical officer of health, and a fairly close acquaintance with the chief Acts of Parliament relating to public health and some other statutes. Some knowledge of the working of logarithms in connection with vital statistics is a useful thing to acquire. During the examination each candidate will be sent to inspect some building or collection of houses, and must submit a formal report thereon.

Courses of instruction for the D.P.H. are now available at a great many centres, and there should be no difficulty in getting in touch with lectures and laboratory accommodation. Most, if not all, the medical schools in London offer the necessary facilities, not only for laboratory work, but also arrange for the outdoor work with a medical officer of health and for the supplementary course of lectures on sanitary law, etc., referred to in Rule 3, Note 2, which is allowed to replace three of the six months to be spent with a medical officer of health. Details of these arrangements, with the names of the lecturers and the co-operating medical officers of health, will be found in the advertisement columns of the medical journals.

A few words may be devoted, in conclusion, to mentioning some of the text-books which have been found most serviceable in preparing for this examination. The list must be regarded as a personal one and not to the prejudice of many other excellent text-books that may be equally useful. The most complete text-book is Notter and Firth's "Theory and Practice of Hygiene" (J. and A. Churchill, 3rd edition, 21s.), but a smaller book, founded on this, is strongly recommended for the student to begin on, and will indeed be found of great value throughout the course; it is published by Longmans, price 4s. 6d. The following list includes a selection of well-tried text-books: Parkes and Kenwood's "Hygiene and Public Health"; Kenwood's "Laboratory Work" (Lewis, 10s. 6d.), Whitelegge and Newman's "Hygiene" (Cassels), Glaister's "Public Health" (Livingstone, 12s.). For bacteriology, Muir and Ritchie (Pentland), Hewlett (Churchill), and Stitt's practical book on bacteria and parasites (Lewis, 6s. 6d.), "The Sanitary Officer's Handbook of Practical Hygiene," by Wanhill and Beveridge (Arnold, 5s.), "Aids to Mathematics of Hygiene," by Ferguson (Baillière, Tindall and Cox, 2s. 6d.), "Sanitary Law and Practice," by Robertson and Porter (Sanitary Publishing Co., 10s. 6d.), "Burnett Hain's Small Book on Sanitary Law," by R. King Brown (Ash and Co., 2s. 6d.).

